# Human parainfluenza virus infection in severe acute respiratory infection cases in Beijing, 2014‐2016: A molecular epidemiological study

**DOI:** 10.1111/irv.12514

**Published:** 2017-11-28

**Authors:** Yang Pan, Yi Zhang, Weixian Shi, Xiaomin Peng, Shujuan Cui, Daitao Zhang, Guilan Lu, Yimeng Liu, Shuangsheng Wu, Peng Yang, Quanyi Wang

**Affiliations:** ^1^ Institute for Infectious Disease and Endemic Disease Control Beijing Center for Disease Prevention and Control (CDC) Beijing China; ^2^ Research Centre for Preventive Medicine of Beijing Beijing China; ^3^ Capital Medical University School of Public Health Beijing China

**Keywords:** hemagglutinin‐neuraminidase, human parainfluenza virus, molecular epidemiology, phylogenetic analysis, severe acute respiratory infection, variation

## Abstract

**Background:**

Severe acute respiratory infection (SARI) threatens human health and even survival, causing a huge number of hospitalized patients every year. However, as one of the most common respiratory viruses circulated worldwide, the epidemiological and phylogenetic characteristics of human parainfluenza virus (HPIV) in these cases were not well known.

**Objectives:**

To reveal the epidemiological features of HPIV infection in SARIs in Beijing area from September 2014 to August 2016.

**Methods:**

A total of 1229 SARI cases in Beijing area were enrolled, investigated, sampled, and tested by multiplex real‐time PCR to identify HPIVs and other common respiratory viruses. Eighteen HPIV‐3 viruses isolated from all HPIV‐positive samples in these SARI cases were sequenced and analyzed.

**Results:**

Among all enrolled cases, 0.81%, 0.73%, 4.48%, and 0.57% were positive for HPIV‐1 to HPIV‐4, respectively. The highest yield rate of HPIV infection occurred in children under 5 years old (9.07%), followed by the patients over 60 years old (6.02%). The phylogenetic information of HPIV‐3 showed that all viruses belonged to Cluster C3a.

**Conclusions:**

Besides the young children, the elders older than 60 years also showed a relatively high infection rate of HPIVs, which should be given comparable attentions. Moreover, the HPIV‐3 circulating in China undergoes continued evolution, suggesting the potential risk of evolved HPIV infection should not be overlooked.

## 1 INTRODUCTION

Human parainfluenza viruses (HPIVs) are enveloped, single‐stranded negative sense RNA viruses that belong to the family Paramyxoviridae. Based on genetic and antigenic variation, HPIVs have been divided into four types: HPIV‐1 to HPIV‐4.^1^ As one of the most common pathogens associated with respiratory tract infections, most children encounter HPIV‐3 within the first 2 years after birth and encounter HPIV‐1 and HPIV‐2 under the age of 5 years old, presenting as upper respiratory tract illness (URTI) or lower respiratory tract illness (LRTI).^2^ For adults, recent studies showed that HPIVs were associated with respiratory tract illness, refractory airway disease, and virus‐induced asthma.^3^, ^4^ These findings point the disease bundle of HPIVs and highlight the need for continuous surveillance of the whole population.

However, our knowledge on HPIVs mainly focused on their infection in children with acute respiratory infections (ARI).^5^, ^6^ Compared with ARIs, severe acute respiratory infection (SARI) cases present a wider distribution and more diverse clinical presentations. Unfortunately, the study on HPIV infection in these cases is very limited. Moreover, virological characteristics of HPIVs in recent years are not fully understood. In our previous study, a SARI surveillance system was built to find influenza infection in these cases. While in this study, a larger number of 1229 SARI cases were enrolled from this surveillance system, screened for HPIVs, and investigated the epidemiological characteristics of infection. Phylogenetic analysis was further performed using the hemagglutinin‐neuraminidase (HN) sequence published previously to reveal the evolution of HPIVs in Beijing area.

## 2 METHODS

### 2.1 Specimens and information collection

Ethical approval for retrospective study was obtained from the institutional review board and human research ethics committee of the Beijing Center for Disease Prevention and Control. Informed consents were obtained from each participant.

This study was conducted in 11 inpatient departments in local hospitals located in urban and suburban districts of Beijing area from September 2014 to August 2016. The enrollment criteria for SARI cases included: (i) inpatients with a temperature >38°C and cough; (ii) onset of clinical symptoms within 10 days.^7^ Nasopharyngeal swabs, throat swabs, or sputum was collected from the enrolled cases. Specimens were stored in 3 mL of virus transport medium at 4°C and tested within 24 hours. Meanwhile, information questionnaires, including the demographic information and vaccine inoculation, were completed by participating physicians at the same time.

### 2.2 Virus detection

Viral RNA was extracted from all specimens using QIAmp Viral Mini Kit (Qiagen, Hilden, Germany) following the manufacturer's instruction. Then, multiplex real‐time PCR kit (Uninovo, Zhenjiang, China) was used to identify HPIVs and other common respiratory viruses (influenza A/B, respiratory syncytial virus, adenovirus, human coronavirus, etc.).

### 2.3 Viral gene sequencing

Eighteen HPIV‐3‐positive specimens were randomly selected and sequenced. Reverse transcription and amplification of HN gene were carried out using the One‐Step RT‐PCR Kit (Qiagen) with primers described previously.^8^ PCR products were selected and purified using EZNA Gel Extraction Kit (Omega, Norcross, GA, USA) and then sequenced by ABI Prism 3130xl automated sequencer (Applied Biosystems, Foster City, CA, USA) (Supporting information 3).

### 2.4 Phylogenetic analyses

In total, 114 representative HN sequences were downloaded from GenBank and used as global background in this study. Nucleotide and deduced amino acid sequences of the HN genes were assembled and aligned using mega software (ver. 6.0.4).^9^ Neighbor‐joining (NJ) phylogeny tree and maximum‐likelihood tree were inferred using mega with Kimura 2‐parameters substitution model and 1000 bootstraps. The nucleotide sequences of the viruses included in this study have been submitted to GenBank (accession numbers: KY355144‐KY355161, Supporting information 3).

### 2.5 Statistical analysis

Data were analyzed using Prism 5 software (GraphPad, La Jolla, CA, USA). Statistical analysis was performed using spss 20.0 (IBM, New York, NY, USA). Difference between groups was evaluated using Pearson's chi‐square or Fisher's exact test, and *P *<* *.05 was considered to be statistically significant.

## 3 RESULTS

A total of 10 595 SARI cases were reported by 11 sentinel hospitals from September 2014 to August 2016. Among all the reported cases, 1229 (11.60%) SARI cases were randomly surveyed, sampled, and tested. Eighty‐one HPIV RNA‐positive cases were identified in this study, including 10 HPIV‐1, 9 HPIV‐2, 55 HPIV‐3, and 7 HPIV‐4 cases, with a yield rate of 0.81%, 0.73%, 4.48%, and 0.57%, respectively. No regional or temporal clustering of HPIV infection was found. Among all 81 HPIV RNA‐positive cases, 18 cases were found to be coinfected with other respiratory viruses. Coinfection with HPIV‐3 and rhinovirus was the most common pattern (Supporting information 1).

**Figure 1:**
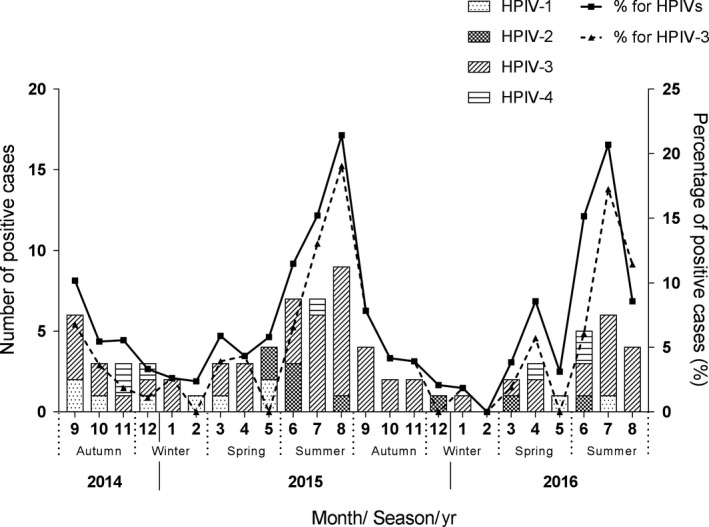
Distribution of the severe acute respiratory infections (SARIs) cases with human parainfluenza virus (HPIV) infection from September 2014 to August 2016

All types of HPIVs were detected every year, except for HPIV‐2 in September to December 2014. Detailed analysis showed that the yield rate of HPIV varied during the year, from the lowest of 0% in February 2016 to the highest of 21.43% in August 2015. As the most common type of HPIVs, HPIV‐3, most of the infection occurred in June to September (66.07%, Figure 1). Moreover, age‐specific infection was proved. Children under 5 years old showed the highest yield rate of HPIV infection (9.07%), and followed by the patients over 60 years old (6.02%). No significant difference was found between HPIV‐positive SARI cases and all SARI cases by gender and hospital stays (*P *=* *.728 and *P *=* *.774, respectively). However, HPIV‐positive SARI cases have a better outcome when compared with other SARI cases (0.24% *vs*. 4.56% for ICU treatment and 0 *vs*. 1.55% for death, Table 1).

**Table 1:** Demographic and clinical characteristics for severe acute respiratory infection (SARI) patients with human parainfluenza virus (HPIV) infection

	Tested SARI cases (%*)	HPIVs				
		Total (%*)	HPIV‐1 (%#)	HPIV‐2 (%#)	HPIV‐3 (%#)	HPIV‐4 (%#)
Collecting time						
September to December 2014	258 (20.99)	15 (5.81)	4 (26.67)	0	8 (53.33)	3 (20.00)
January to December 2015	654 (53.21)	45 (6.88)	4 (8.89)	7 (15.56)	33 (73.33)	1 (2.22)
January to August 2016	317 (25.79)	21 (6.62)	2 (9.52)	2 (9.52)	14 (66.67)	3 (14.29)
Gender						
Male	724 (58.91)	46 (6.35)	6 (13.04)	5 (10.87)	30 (65.22)	5 (10.87)
Female	505 (41.09)	35 (6.93)	4 (11.43)	4 (11.43)	25 (71.43)	2 (5.71)
Age (y)						
0‐5	375 (30.51)	34 (9.07)	5 (14.71)	4 (11.76)	22 (64.71)	3 (8.82)
6‐15	157 (12.77)	8 (5.10)	0	2 (25.00)	4 (50.00)	2 (20.00)
16‐25	31 (2.52)	1 (3.23)	0	0	1 (100.00)	0
26‐60	251 (20.42)	13 (5.18)	3 (23.08)	2 (15.38)	8 (61.54)	0
61‐	415 (33.77)	25 (6.02)	2 (8.00)	1 (4.00)	20 (80.00)	2 (8.00)
Hospital stays (wk)						
1	490 (39.87)	34 (6.94)	6 (17.65)	3 (8.82)	22 (64.71)	3 (8.82)
1‐2	452 (36.78)	26 (5.75)	4 (15.38)	3 (11.54)	17 (65.38)	2 (7.69)
2‐4	195 (15.87)	13 (6.67)	0	2 (15.38)	11 (84.62)	0
4	92 (7.49)	8 (8.70)	0	1 (12.50)	5 (62.50)	2 (25.00)
Outcome						
ICU treatment	56 (4.56)	3 (0.24)	0	0	3 (100.00)	0
Death	19 (1.55)	0	0	0	0	0
Total	1229	81 (6.59)	10 (12.35)	9 (11.11)	55 (67.90)	7 (8.64)

*In all cases, #In cases for each category.

A total of 18 full sequences of HN gene (1719 nt)‐derived HPIV‐3 virus in SARI cases were analyzed in this study, including 3 sequences in 2014, 10 sequences in 2015, and 5 sequences in 2016. The nucleotide sequence and deduced amino acid homologies among these viruses were between 98.84%‐100% and 99.65%‐100%, respectively, which suggested high genetic conservation of HPIV‐3 in Beijing area. No significant difference between viruses derived from patients with different age or seasonality was observed. Compared to the prototype strain Wash/47885/57, 15 amino acid substitutions (M21T, I40T, I53T, H62R, I76V, M82V, M118I, L138P, K168R, V191I, I391V, V348A, I522R, R524K, and S527P) were found in the tested HPIV‐3 viruses. Among them, K168R and V191I were first identified in Asia.

**Figure 2:**
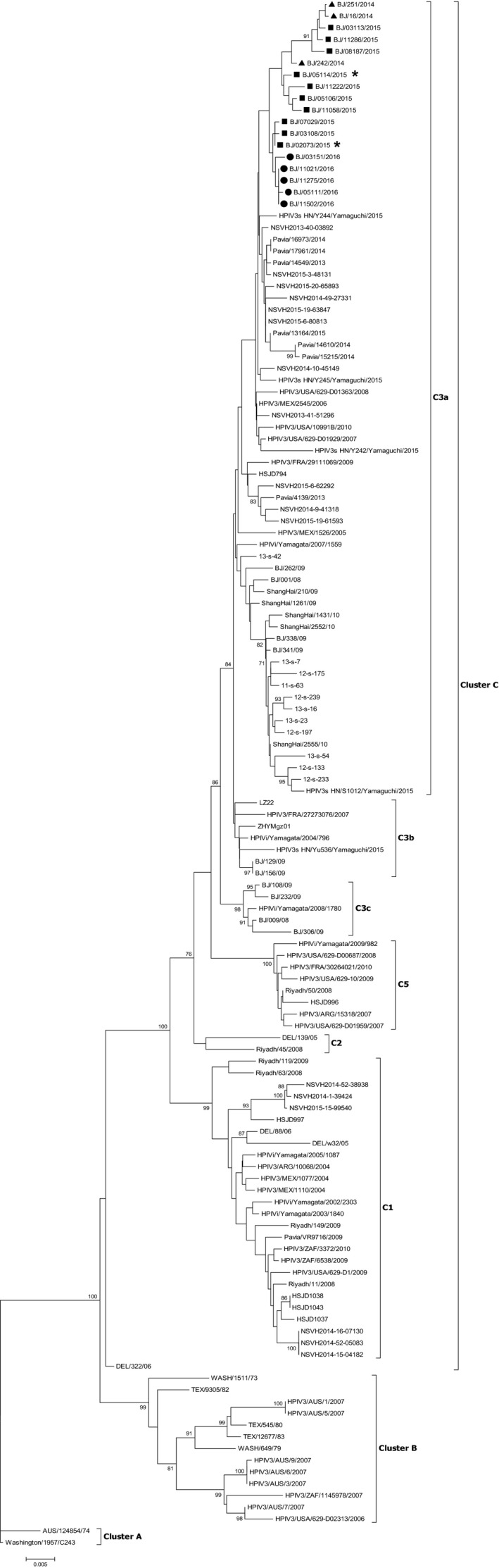
Phylogenetic analysis of hemagglutinin‐neuraminidase (HN) gene of human parainfluenza virus 3 from severe acute respiratory infection (SARI) cases. The phylogenetic trees were constructed using mega program and employing Neighbor‐joining (NJ) method with Kimura 2‐parameters substitution model and 1000 bootstraps. Only bootstrap number >70% is shown. ▲, ■, and ●, the viruses isolated in 2014, 2015, and 2016, respectively. *, The representative HN sequences in 2014‐2016 in Beijing

We further analyzed the sequences of HN gene of tested viruses and circulating strains worldwide and then evaluated the phylogenetic relationship among them. All the viruses belonged to Cluster C3a, which was the major circulating cluster in Asia. However, the viruses can be further distinguished into two C3a‐related group, BJ/05114/2015‐like viruses and BJ/02073/2015‐like viruses, with at least 0.004 nucleotide divergence between them. Moreover, these two group viruses co‐circulated in Beijing area before the middle of 2015. Then a genetic shift occurred, leading to a dominate role of BJ/02073/2015‐like viruses. No BJ/05114/2015‐like viruses were identified in 2016 (Figure 2, Supporting information 2).

## 4 DISCUSSION

In the present study, 1229 SARI patients were enrolled, and the HPIVs in these cases were first investigated. Previous studies were usually performed in ARI cases, especially in outpatients.^10^, ^11^ Noteworthily, compared with the definition of ARI (presence of constitutional signs or symptom of respiratory tract infection, ie, cough and fast breathing), the criteria of SARI puts more focus on the hospitalized severe cases. As expected, infants and young child <5 years old showed the highest infection rate of HPIVs in all SARI cases, which concur with the studies carried out in ARIs. However, the high yield rate of HPIVs in elder population was observed: 6.02% (25/415) of SARI cases over 60 years old were HPIV‐positive, which was higher than that in mild ARI cases.^11^ Although the HPIV infection usually presents as mild and self‐limiting illness, and no ICU‐treated cases and death were reported in this study, elder patients still bear a high risk in HPIV infection, due to their complex underlying diseases and relatively low immune response. Moreover, we found that most HIPV infection occurred in June to September (58.54%), which showed a relatively delayed epidemiological peak compared with the study in early years in the same area (March‐August).^8^

An important finding of this study was the genetic characteristic of current circulating HPIV‐3 viruses. Two major surface glycoproteins, the HN and the fusion (F) proteins, are essential for virus assembly.^1^ In particular, HN glycoprotein regulates the interaction between virus and host cells and possesses the largest antigenicity in host immunity against HPIV infection(12). Several antigenic epitopes of the HN glycoprotein have been previously characterized.^12^, ^13^ Three amino acid substitutions in antigenic epitopes were found in this study, including K168R (16.67%), H295Y (100%), and I391V (100%). In particular, the K168R mutation locates very close to amino acids of significant importance to the integrity of distinct HN epitopes (residues 171),^14^ and it has not been identified in Asia before. Although these variants become non‐dominant since the middle of 2015, the potential antigenic drift due to the substitutions should be further evaluated.

The phylogenetic analysis showed that HPIV‐3 Cluster C remained as the most dynamic and widespread group worldwide, which concur with previous studies.^6^, ^15^ However, the viruses tested in this study were composed by two subgroups, BJ/05114/2015‐like viruses and BJ/02073/2015‐like viruses. The former possesses some genetic characteristic derived from European strains, such as K168R, while the latter was much more like the strains circulated in China and Japan. These results suggested the complex evolution of HPIV‐3 both related temporally and geographically.

In conclusion, the common infection of HPIVs in SARI cases is reported in this study. The HPIVs in young children, as well as the elders older than 60 years, should be paid more attention. The phylogenetic information of the most common HPIVs, HPIV‐3, was also shown here. The continual evolution and genetic diversity of HPIV‐3 highlight the needs for more long‐term investigation both in China and around the world in the future.

## References

[1] Henrickson KJ . Parainfluenza viruses. Clin Microbiol Rev. 2003;16:242‐264. 12692097 10.1128/CMR.16.2.242-264.2003 PMC153148

[2] Hall CB . Respiratory syncytial virus and parainfluenza virus. N Engl J Med. 2001;344:1917‐1928. 11419430 10.1056/NEJM200106213442507

[3] Tsukagoshi H , Ishioka T , Noda M , et al. Molecular epidemiology of respiratory viruses in virus‐induced asthma. Front Microbiol. 2013;4:278. 24062735 10.3389/fmicb.2013.00278 PMC3771312

[4] Rosenthal LA , Avila PC , Heymann PW , et al. Viral respiratory tract infections and asthma: the course ahead. J Allergy Clin Immunol. 2010;125:1212‐1217. 20513518 10.1016/j.jaci.2010.04.002 PMC2880817

[5] Liu WK , Liu Q , Chen DH , et al. Epidemiology and clinical presentation of the four human parainfluenza virus types. BMC Infect Dis. 2013;13:28. 23343342 10.1186/1471-2334-13-28 PMC3560251

[6] Tsutsui R , Tsukagoshi H , Nagasawa K , et al. Genetic analyses of the fusion protein genes in human parainfluenza virus types 1 and 3 among patients with acute respiratory infections in Eastern Japan from 2011 to 2015. J Med Microbiol. 2017;66:160‐168. 28266286 10.1099/jmm.0.000431

[7] World Health Organization . WHO global epidemiological surveillance standards for influenza 2014. http://www.who.int/influenza/resources/documents/influenza_surveillance_manual/en/. Accessed September 2, 2017.

[8] Mao N , Ji Y , Xie Z , et al. Human parainfluenza virus‐associated respiratory tract infection among children and genetic analysis of HPIV‐3 strains in Beijing, China. PLoS One. 2012;7:e43893. 22937119 10.1371/journal.pone.0043893 PMC3429441

[9] Tamura K , Stecher G , Peterson D , et al. MEGA6: molecular evolutionary genetics analysis version 6.0. Mol Biol Evol. 2013;30:2725‐2729. 24132122 10.1093/molbev/mst197 PMC3840312

[10] Mizuta K , Tsukagoshi H , Ikeda T , et al. Molecular evolution of the haemagglutinin‐neuraminidase gene in human parainfluenza virus type 3 isolates from children with acute respiratory illness in Yamagata prefecture, Japan. J Med Microbiol. 2014;63(Pt 4):570‐577. 24464692 10.1099/jmm.0.068189-0

[11] Weixian S , Cui S , Gong C , et al. Prevalence of human parainfluenza virus in patients with acute respiratory tract infections in Beijing, 2011‐2014. Influenza Other Respir Viruses. 2015;9:305‐307. 10.1111/irv.12336 PMC4605411 26230490

[12] Lawrence MC , Borg NA , Streltsov VA , et al. Structure of the haemagglutinin‐neuraminidase from human parainfluenza virus type III. J Mol Biol. 2004;335:1343‐1357. 14729348 10.1016/j.jmb.2003.11.032

[13] Godoy C , Peremiquel‐Trillas P , Andres C , et al. A molecular epidemiological study of human parainfluenza virus type 3 at a tertiary university hospital during 2013‐2015 in Catalonia, Spain. Diagn Microbiol Infect Dis. 2016;86:153‐159. 27524509 10.1016/j.diagmicrobio.2016.07.023 PMC7127006

[14] van Wyke Coelingh KL , Winter CC , Jorgensen ED , et al. Antigenic and structural properties of the hemagglutinin‐neuraminidase glycoprotein of human parainfluenza virus type 3: sequence analysis of variants selected with monoclonal antibodies which inhibit infectivity, hemagglutination, and neuraminidase activities. J Virol. 1987;61:1473‐1477. 2437318 10.1128/jvi.61.5.1473-1477.1987 PMC254125

[15] Almajhdi FN . Hemagglutinin‐neuraminidase gene sequence‐based reclassification of human parainfluenza virus 3 variants. Intervirology. 2015;58:35‐40. 25592955 10.1159/000369208

