# Supplementary material for: Human parainfluenza virus infection in severe acute respiratory infection cases in Beijing, 2014‐2016: A molecular epidemiological study
Source: Influenza Other Respir Viruses. 2017 Nov 28;11(6):564–8. doi: 10.1111/irv.12514 (PMC5705688; doi:10.1111/irv.12514)
Supplement: Supplementary file 1 [file IRV-11-564-s001.docx]

**Supporting information 1** The coinfection of HPIVs and other respiratory pathogens

Table 1 The coinfection of HPIVs and other respiratory pathogens

| Coinfection | No. of cases | | | | Total no. of cases |
| --- | --- | --- | --- | --- | --- |
|  | HPIV-1 | HPIV-2 | HPIV-3 | HPIV-4 |  |
| HPIV, HRV |  |  | 6 | 1 | 7 |
| HPIV, RSV | 1 |  | 2 |  | 3 |
| HPIV, FluB |  |  | 2 |  | 2 |
| HPIV, ADV | 1 | 1 |  |  | 2 |
| HPIV, HCoV |  |  | 2 |  | 2 |
| HPIV, FluA |  |  | 1 |  | 1 |
| HPIV, HRV, FluA |  |  | 1 |  | 1 |
| Total | 2 | 1 | 14 | 1 | 18 |

HPIV, Human parainfluenza virus, HRV, Human parainfluenza virus, RSV, Respiratory syncytial virus, FluB, Influenza B virus, ADV, Adenovirus, HCoV, Human coronavirus, FluA, Influenza A virus.

**Supporting information 2** Phylogentic analysis of HN gene of human parainfluenza virus 3 from SARI cases by Maximum-likelihood method.


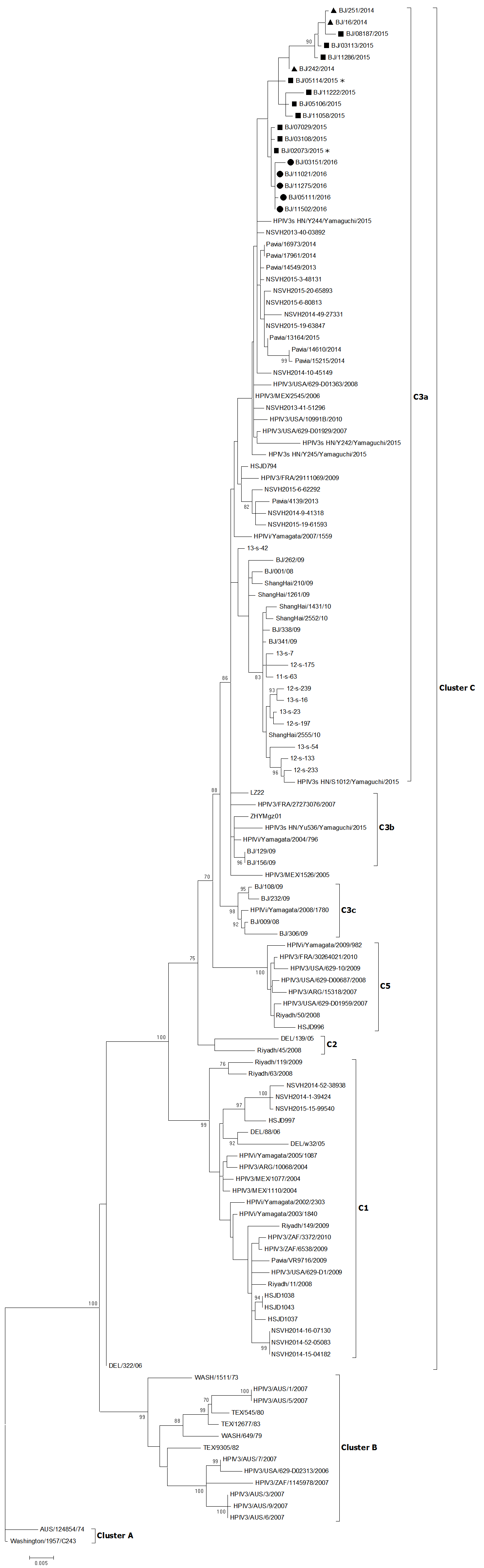


The phylogenetic trees were constructed using MEGA program and employing Maximum-likelihood (ML) method with Kimura 2-parameters substitution model and 1,000 bootstraps. Only bootstrap number > 70% is shown. ▲, ■ and ●, the viruses isolated in 2014, 2015 and 2016, respectively. *, The representative HN sequences in 2014-2016 in Beijing.

**Supporting information 3** Technical Appendix

# Methods

## Specimens and information collection

This study was performed in 11 inpatient departments in local hospitals located in urban and suburban districts of Beijing area from Sep 2014 to Aug 2016. The hospitals were listed as follows: Beijing Tiantan Hospital, Beijing Huaxin Hospital, Beijing Shijingshan Hospital, Peking University People’s Hospital, Aerospace Center Hospital, People’s Hospital of Beijing Daxing District, Beijing Miyun Hospital, Beijing Changping Hospital, Beijing Jingmei Group General Hospital, Beijing Huairou Hospital and Beijing Liangxiang Hospital.

## Gene sequencing

18 HPIV-3 positive specimens were randomly selected and sequenced. Viral RNA was extracted from all specimens using QIAmp Viral Mini Kit (Qiagen, Hilden, Germany) following the manufacturer’s instruction. Then reverse transcription and ampliﬁcation of hemagglutinin-neuraminidase (HN) gene were carried out using the One-Step RT-PCR Kit (Qiagen) following the manufacturer’s instruction with primers described previously(Mao N, et al. 2012) (Technical Appendix Table 1). 5μl viral RNA was added as in a 50μl reaction volume as template. The cycling conditions were as follows: an initial reverse transcription at 50℃ for 30 min, an initial PCR activation at 95℃for 15 min, followed by 35 cycles of 3-step cycling (94℃ for 1 min, 50℃ for 1 min and 72℃ for 2 min), and a final extension of 10 min at 72℃. PCR products were analyzed and purified by EZNA Gel Extraction Kit (Omega, Norcross, USA), then sequenced by ABI Prism 3130xl automated sequencer with sequencing primers (Applied Biosystems, Foster City, USA) (Technical Appendix Table 1).

## Phylogenetic analyses

114 representative HN sequences were downloaded from GenBank and used as global background in this study (Technical Appendix Table 2). Nucleotide and deduced amino acid sequences of the HN genes were assembled and aligned using MEGA software (ver. 6.0.4) (Tamura K, et al. 2013). Neighbor-joining (NJ) phylogeny tree and Maximum-likelihood (ML) tree were inferred by using MEGA with Kimura 2-parameters substitution model and 1,000 bootstraps. The nucleotide sequences of the viruses included in this study have been submitted to GenBank (accession numbers: KY355144- KY355161).

Technical Appendix Table 1

The primers used in sequencing

| RT-PCR primers | 3HNn+ | ATGGAATACTGGAAGCACACCAACCAC |
| --- | --- | --- |
|  | 3HNn- | TATCTCGAGTTATGATTAACTGCAGC |
| Sequencing primers | 3HNn+ | ATGGAATACTGGAAGCACACCAACCAC |
|  | P3- | CTGAATTGTAAGAAGCCTTGT |
|  | PF+ | CTCGAGGTTGTCAGGATATAG |
|  | P5+ | AACTGTGTTCAACTCCCAAAG |
|  | P6+ | CAAGTTGGCATAGCAAGTTAC |

Designed by Mao N, et al. 2012

Technical Appendix Table 2

| Accession No. | Description |
| --- | --- |
| AB623440 | Human parainfluenza virus 3 HN gene for hemagglutinin-neuraminidase, partial cds, strain: HPIVi/Yamagata/2002/2303 |
| AB623458 | Human parainfluenza virus 3 HN gene for hemagglutinin-neuraminidase, partial cds, strain: HPIVi/Yamagata/2003/1840 |
| AB623459 | Human parainfluenza virus 3 HN gene for hemagglutinin-neuraminidase, partial cds, strain: HPIVi/Yamagata/2004/796 |
| AB623480 | Human parainfluenza virus 3 HN gene for hemagglutinin-neuraminidase, partial cds, strain: HPIVi/Yamagata/2005/1087 |
| AB623577 | Human parainfluenza virus 3 HN gene for hemagglutinin-neuraminidase, partial cds, strain: HPIVi/Yamagata/2007/1559 |
| AB623626 | Human parainfluenza virus 3 HN gene for hemagglutinin-neuraminidase, partial cds, strain: HPIVi/Yamagata/2008/1780 |
| AB623628 | Human parainfluenza virus 3 HN gene for hemagglutinin-neuraminidase, partial cds, strain: HPIVi/Yamagata/2009/982 |
| EU326526 | Human parainfluenza virus 3 strain ZHYMgz01, complete genome |
| EU814623 | Human parainfluenza virus 3 strain DEL/322/06 hemagglutinin-neuraminidase (HN) gene, complete cds |
| EU814624 | Human parainfluenza virus 3 strain DEL/88/06 hemagglutinin-neuraminidase (HN) gene, complete cds |
| EU814625 | Human parainfluenza virus 3 strain DEL/w32/05 hemagglutinin-neuraminidase (HN) gene, complete cds |
| EU814626 | Human parainfluenza virus 3 strain DEL/139/05 hemagglutinin-neuraminidase (HN) gene, complete cds |
| FJ455842 | Human parainfluenza virus 3 strain LZ22, complete genome |
| GU732130 | Human parainfluenza virus 3 strain BJ/001/08 hemagglutinin-neuraminidase (HN) gene, complete cds |
| GU732134 | Human parainfluenza virus 3 strain BJ/009/08 hemagglutinin-neuraminidase (HN) gene, complete cds |
| GU732142 | Human parainfluenza virus 3 strain BJ/108/09 hemagglutinin-neuraminidase (HN) gene, complete cds |
| GU732143 | Human parainfluenza virus 3 strain BJ/129/09 hemagglutinin-neuraminidase (HN) gene, complete cds |
| GU732145 | Human parainfluenza virus 3 strain BJ/156/09 hemagglutinin-neuraminidase (HN) gene, complete cds |
| GU732149 | Human parainfluenza virus 3 strain BJ/232/09 hemagglutinin-neuraminidase (HN) gene, complete cds |
| GU732154 | Human parainfluenza virus 3 strain BJ/262/09 hemagglutinin-neuraminidase (HN) gene, complete cds |
| GU732160 | Human parainfluenza virus 3 strain BJ/306/09 hemagglutinin-neuraminidase (HN) gene, complete cds |
| GU732170 | Human parainfluenza virus 3 strain BJ/338/09 hemagglutinin-neuraminidase (HN) gene, complete cds |
| GU732171 | Human parainfluenza virus 3 strain BJ/341/09 hemagglutinin-neuraminidase (HN) gene, complete cds |
| HM460886 | Human parainfluenza virus 3 strain Riyadh 11/2008 hemagglutinin-neuramindase (HN) gene, complete cds |
| HM460887 | Human parainfluenza virus 3 strain Riyadh 149/2009 hemagglutinin-neuramindase (HN) gene, complete cds |
| JN089924 | Human parainfluenza virus 3 strain Washington/1957 C243 hemagglutinin-neuraminidase mRNA, complete cds |
| JX131646 | Human parainfluenza virus 3 strain Riyadh 45/2008 hemagglutinin-neuramindase glycoprotein gene, complete cds |
| JX131647 | Human parainfluenza virus 3 strain Riyadh 50/2008 hemagglutinin-neuramindase glycoprotein gene, complete cds |
| JX131648 | Human parainfluenza virus 3 strain Riyadh 119/2009 hemagglutinin-neuramindase glycoprotein gene, complete cds |
| JX131649 | Human parainfluenza virus 3 strain Riyadh 63/2008 hemagglutinin-neuramindase glycoprotein gene, complete cds |
| KF217153 | Human parainfluenza virus 3 isolate HSJD1037 hemagglutinin-neuraminidase (HN) gene, partial cds |
| KF217154 | Human parainfluenza virus 3 isolate HSJD1038 hemagglutinin-neuraminidase (HN) gene, partial cds |
| KF217155 | Human parainfluenza virus 3 isolate HSJD1043 hemagglutinin-neuraminidase (HN) gene, partial cds |
| KF217168 | Human parainfluenza virus 3 isolate HSJD794 hemagglutinin-neuraminidase (HN) gene, partial cds |
| KF217169 | Human parainfluenza virus 3 isolate HSJD996 hemagglutinin-neuraminidase (HN) gene, partial cds |
| KF217170 | Human parainfluenza virus 3 isolate HSJD997 hemagglutinin-neuraminidase (HN) gene, partial cds |
| KF530225 | Human parainfluenza virus 3 strain HPIV3/AUS/9/2007, complete genome |
| KF530226 | Human parainfluenza virus 3 strain HPIV3/AUS/1/2007, complete genome |
| KF530230 | Human parainfluenza virus 3 strain HPIV3/AUS/7/2007, complete genome |
| KF530232 | Human parainfluenza virus 3 strain HPIV3/USA/10991B/2010, complete genome |
| KF530233 | Human parainfluenza virus 3 strain HPIV3/FRA/29111069/2009, complete genome |
| KF530234 | Human parainfluenza virus 3 strain HPIV3/MEX/1526/2005, complete genome |
| KF530236 | Human parainfluenza virus 3 strain HPIV3/FRA/30264021/2010, complete genome |
| KF530241 | Human parainfluenza virus 3 strain HPIV3/USA/629-D1/2009, complete genome |
| KF530242 | Human parainfluenza virus 3 strain HPIV3/USA/629-D00687/2008, complete genome |
| KF530243 | Human parainfluenza virus 3 strain HPIV3/AUS/3/2007, complete genome |
| KF530245 | Human parainfluenza virus 3 strain HPIV3/ARG/10068/2004, complete genome |
| KF530247 | Human parainfluenza virus 3 strain HPIV3/USA/629-D02313/2006, complete genome |
| KF530249 | Human parainfluenza virus 3 strain HPIV3/AUS/6/2007, complete genome |
| KF530250 | Human parainfluenza virus 3 strain HPIV3/MEX/2545/2006, complete genome |
| KF530251 | Human parainfluenza virus 3 strain HPIV3/AUS/5/2007, complete genome |
| KF530252 | Human parainfluenza virus 3 strain HPIV3/USA/629-D01363/2008, complete genome |
| KF530253 | Human parainfluenza virus 3 strain HPIV3/FRA/27273076/2007, complete genome |
| KF530256 | Human parainfluenza virus 3 strain HPIV3/USA/629-D01929/2007, complete genome |
| KF530257 | Human parainfluenza virus 3 strain HPIV3/ARG/15318/2007, complete genome |
| KF600603 | Human parainfluenza virus 3 strain ShangHai/210/09 hemagglutinin-neuraminidase (HN) gene, complete cds |
| KF600604 | Human parainfluenza virus 3 strain ShangHai/1261/09 hemagglutinin-neuraminidase (HN) gene, complete cds |
| KF600605 | Human parainfluenza virus 3 strain ShangHai/1431/10 hemagglutinin-neuraminidase (HN) gene, complete cds |
| KF600606 | Human parainfluenza virus 3 strain ShangHai/2552/10 hemagglutinin-neuraminidase (HN) gene, complete cds |
| KF600607 | Human parainfluenza virus 3 strain ShangHai/2555/10 hemagglutinin-neuraminidase (HN) gene, complete cds |
| KF687317 | Human parainfluenza virus 3 strain HPIV3/USA/629-10/2009, complete genome |
| KF687318 | Human parainfluenza virus 3 strain HPIV3/USA/629-D01959/2007, complete genome |
| KF687319 | Human parainfluenza virus 3 strain HPIV3/MEX/1077/2004, complete genome |
| KF687321 | Human parainfluenza virus 3 strain HPIV3/MEX/1110/2004, complete genome |
| KF687336 | Human parainfluenza virus 3 strain HPIV3/ZAF/1145978/2007, complete genome |
| KF687340 | Human parainfluenza virus 3 strain HPIV3/ZAF/3372/2010, complete genome |
| KF687346 | Human parainfluenza virus 3 strain HPIV3/ZAF/6538/2009, complete genome |
| KP690747 | Human parainfluenza virus 3 strain 11-s-63 hemagglutinin-neuraminidase (HN) gene, complete cds |
| KP690749 | Human parainfluenza virus 3 strain 12-s-133 hemagglutinin-neuraminidase (HN) gene, complete cds |
| KP690761 | Human parainfluenza virus 3 strain 12-s-233 hemagglutinin-neuraminidase (HN) gene, complete cds |
| KP690762 | Human parainfluenza virus 3 strain 12-s-239 hemagglutinin-neuraminidase (HN) gene, complete cds |
| KP690769 | Human parainfluenza virus 3 strain 13-s-7 hemagglutinin-neuraminidase (HN) gene, complete cds |
| KP690770 | Human parainfluenza virus 3 strain 13-s-16 hemagglutinin-neuraminidase (HN) gene, complete cds |
| KP690771 | Human parainfluenza virus 3 strain 13-s-23 hemagglutinin-neuraminidase (HN) gene, complete cds |
| KP690777 | Human parainfluenza virus 3 strain 12-s-197 hemagglutinin-neuraminidase (HN) gene, complete cds |
| KP690791 | Human parainfluenza virus 3 strain 13-s-42 hemagglutinin-neuraminidase (HN) gene, complete cds |
| KP690792 | Human parainfluenza virus 3 strain 13-s-54 hemagglutinin-neuraminidase (HN) gene, complete cds |
| KP690793 | Human parainfluenza virus 3 strain 12-s-175 hemagglutinin-neuraminidase (HN) gene, complete cds |
| KT796368 | Human parainfluenza virus 3 strain NSVH2013-40-03892 haemagglutinin-neuraminidase (HN) gene, complete cds |
| KT796369 | Human parainfluenza virus 3 strain NSVH2013-41-51296 haemagglutinin-neuraminidase (HN) gene, complete cds |
| KT796370 | Human parainfluenza virus 3 strain NSVH2014-9-41318 haemagglutinin-neuraminidase (HN) gene, complete cds |
| KT796371 | Human parainfluenza virus 3 strain NSVH2014-10-45149 haemagglutinin-neuraminidase (HN) gene, complete cds |
| KT796423 | Human parainfluenza virus 3 strain NSVH2014-49-27331 haemagglutinin-neuraminidase (HN) gene, complete cds |
| KT796424 | Human parainfluenza virus 3 strain NSVH2014-52-38938 haemagglutinin-neuraminidase (HN) gene, complete cds |
| KT796425 | Human parainfluenza virus 3 strain NSVH2014-1-39424 haemagglutinin-neuraminidase (HN) gene, complete cds |
| KT796426 | Human parainfluenza virus 3 strain NSVH2015-3-48131 haemagglutinin-neuraminidase (HN) gene, complete cds |
| KT796431 | Human parainfluenza virus 3 strain NSVH2015-6-80813 haemagglutinin-neuraminidase (HN) gene, complete cds |
| KT796432 | Human parainfluenza virus 3 strain NSVH2015-6-62292 haemagglutinin-neuraminidase (HN) gene, complete cds |
| KT796454 | Human parainfluenza virus 3 strain NSVH2015-19-63847 haemagglutinin-neuraminidase (HN) gene, complete cds |
| KT796455 | Human parainfluenza virus 3 strain NSVH2015-20-65893 haemagglutinin-neuraminidase (HN) gene, complete cds |
| KT796457 | Human parainfluenza virus 3 strain NSVH2014-16-07130 haemagglutinin-neuraminidase (HN) gene, complete cds |
| KT796458 | Human parainfluenza virus 3 strain NSVH2014-52-05083 haemagglutinin-neuraminidase (HN) gene, complete cds |
| KT796459 | Human parainfluenza virus 3 strain NSVH2015-15-99540 haemagglutinin-neuraminidase (HN) gene, complete cds |
| KT796460 | Human parainfluenza virus 3 strain NSVH2014-15-04182 haemagglutinin-neuraminidase (HN) gene, complete cds |
| KT796461 | Human parainfluenza virus 3 strain NSVH2015-19-61593 haemagglutinin-neuraminidase (HN) gene, complete cds |
| KT898925 | Human parainfluenza virus 3 strain Pavia/4139/2013 hemagglutinin-neuraminidase (HN) gene, complete cds |
| KT898926 | Human parainfluenza virus 3 strain Pavia/14549/2013 hemagglutinin-neuraminidase (HN) gene, complete cds |
| KT898927 | Human parainfluenza virus 3 strain Pavia/VR9716/2009 hemagglutinin-neuraminidase (HN) gene, complete cds |
| KT898928 | Human parainfluenza virus 3 strain Pavia/13164/2015 hemagglutinin-neuraminidase (HN) gene, complete cds |
| KT898929 | Human parainfluenza virus 3 strain Pavia/14610/2014 hemagglutinin-neuraminidase (HN) gene, complete cds |
| KT898930 | Human parainfluenza virus 3 strain Pavia/16973/2014 hemagglutinin-neuraminidase (HN) gene, complete cds |
| KT898931 | Human parainfluenza virus 3 strain Pavia/17961/2014 hemagglutinin-neuraminidase (HN) gene, complete cds |
| KT898932 | Human parainfluenza virus 3 strain Pavia/15215/2014 hemagglutinin-neuraminidase (HN) gene, complete cds |
| LC102231 | Human parainfluenza virus 3 HN gene for hemagglutinin-neuraminidase, partial cds, strain: HPIV3s_HN/S1012/Yamaguchi/2015 |
| LC102232 | Human parainfluenza virus 3 HN gene for hemagglutinin-neuraminidase, partial cds, strain: HPIV3s_HN/Y242/Yamaguchi/2015 |
| LC102233 | Human parainfluenza virus 3 HN gene for hemagglutinin-neuraminidase, partial cds, strain: HPIV3s_HN/Y244/Yamaguchi/2015 |
| LC102234 | Human parainfluenza virus 3 HN gene for hemagglutinin-neuraminidase, partial cds, strain: HPIV3s_HN/Y245/Yamaguchi/2015 |
| LC102235 | Human parainfluenza virus 3 HN gene for hemagglutinin-neuraminidase, partial cds, strain: HPIV3s_HN/Yu536/Yamaguchi/2015 |
| PIFHNA | Human parainfluenza virus type 3 WASH/1511/73 hemagglutinin- neuraminidase (HN) mRNA, complete cds |
| PIFHNBA | Human parainfluenza virus type 3 AUS/124854/74 hemagglutinin- neuraminidase (HN) mRNA, complete cds |
| PIFHNC | Human parainfluenza virus type 3 WASH/649/79 hemagglutinin- neuraminidase (HN) mRNA, complete cds |
| PIFHND | Human Human parainfluenza virus type 3 TEX/545/80 hemagglutinin- neuraminidase (HN) mRNA, complete cds |
| PIFHNE | Human parainfluenza virus type 3 TEX/9305/82 hemagglutinin- neuraminidase (HN) mRNA, complete cds |
| PIFHNF | Human parainfluenza virus type 3 TEX/12677/83 hemagglutinin- neuraminidase (HN) mRNA, complete cds |
